# The Impact of Niclosamide Exposure on the Activity of Antioxidant Enzymes and the Expression of Glucose and Lipid Metabolism Genes in Black Carp (*Mylopharyngodon piceus*)

**DOI:** 10.3390/genes14122196

**Published:** 2023-12-10

**Authors:** Hao Wu, Xiping Yuan, Min Xie, Jinwei Gao, Zhenzhen Xiong, Rui Song, Zhonggui Xie, Dongsheng Ou

**Affiliations:** Hunan Fisheries Science Institute, Changsha 410153, China; wh17380133463@163.com (H.W.); kerryuan@163.com (X.Y.); xieminhaha@126.com (M.X.); gaojinwei163@163.com (J.G.); 13763092902@163.com (Z.X.); xieice123@163.com (Z.X.); njdodx@163.com (D.O.)

**Keywords:** niclosamide, oxidative stress, metabolic disorder, barrier functions, black carp

## Abstract

Niclosamide (NIC, 2′,5-dichloro-4′-nitrosalicylanilide) is a salicylanilide molluscicide, and the extensive utilization and environmental pollution associated with NIC engender a potential hazard to both human health and the wellbeing of aquatic organisms. However, the mechanism of the chronic toxicity of NIC at environmentally relevant concentrations in terms of oxidative stress, metabolic disorder, and barrier functions in black carp (*Mylopharyngodon piceus*) is unknown. Therefore, healthy juvenile black carp (*M. piceus*) (average weight: 38.2 ± 2.5 g) were exposed to NIC at an environmentally realistic concentration (0, 10, and 50 μg/L) for 28 days. The findings of this study indicate that exposure to NIC resulted in reductions in weight gain, decreased activity of antioxidant enzymes, and increased expression of the Nrf2 gene. Furthermore, the liver demonstrated a greater accumulation of NIC than that in the gut and gills, as determined with a chemical analysis. Additionally, NIC exposure led to a significant reduction in ATP content and the activity of Na^+^/K^+^-ATPase and Ca^2+^/Mg^2+^-ATPase in the gut. Meanwhile, exposure to NIC resulted in a decrease in the liver glucose (Glu) level, gut cholesterol (CHO), and glycogen (Gln) and triglyceride (TG) content in all examined tissues. Conversely, it led to an increase in tissue lactic acid (LA) and acetyl-CoA levels, as well as LDH activity. Furthermore, NIC exposure at environmentally relevant concentrations demonstrated an upregulation in the expression of genes associated with glycolysis, such as *PK* and *GK*, while concurrently downregulating the gluconeogenesis gene *G6Pase*. Additionally, NIC exhibited an upregulation in the expression of genes related to *β*-oxidation, such as *CPT1* and *ACOX*, while downregulating genes involved in triglyceride synthesis, including *SREBP1*, *GPAT*, *FAS*, and *ACC1*. Moreover, NIC facilitated fatty acid transportation through the overexpression of *FATP* and *Fat/cd36*. These results suggest that chronic exposure to NIC is associated with oxidative stress, compromised barrier function, and metabolic disorder. Moreover, these results underscore the significance of assessing the potential consequences of NIC for black carp and aquatic environments for aquaculture.

## 1. Introduction

In the early 1960s, an available chemosynthetic anthelmintic with the code name Bayer73 was first synthesized, and today, it is called niclosamide (NIC, C_13_H_8_C_l2_N_2_O_4_) [1]. This drug is a yellow powder and is comfortably soluble in hot ethanol and cyclohexanone, which results in the formation of stable compounds. These compounds were declared as the only commercially available molluscicides by the World Health Organization (WHO), which have been widely used to eliminate snails and reduce the prevalence of schistosomiasis since 1972 [2]. Historically, early studies discovered that an NIC solution of approximately 1.0 g/m^2^ achieved the best molluscicidal effects for snail control through either immersion or spraying [3]. At the same time, as a good anthelmintic drug and a lampricide, NIC has been extensively used in agriculture and aquaculture for nearly 40 years, and it is also suitable for the treatment of a variety of diseases [1,4,5,6]. In China, the chemical molluscicide known as NIC has been identified as the most extensively utilized and enduring substance. Recent studies have revealed that the presence of NIC residues has resulted in widespread environmental contamination and poses a potential risk to public health. This is primarily attributed to the substantial annual consumption of NIC and its resistance to hydrolysis [2,7,8]. For example, in the Poyang Lake of China, NIC concentrations were up to 38 μg/L and 474 μg/kg in surface water and sediment samples, respectively [9]. Additionally, in other lentic areas, such as the Great Lakes, more than 1800 kg of NIC were released into the aqueous system in 2020 [10]. While the utilization of this NIC-based molluscicide and lampricide yields a notable decline in snail and lamprey populations [4,11], it concurrently leads to the demise and decomposition of various nontarget aquatic organisms, including fish [7] and reptilian species [12].

Once in an aquatic system, NIC is ingested by aquatic organisms through the processes of bioaccumulation and biomagnification, subsequently affecting nontarget aquatic species and causing toxic effects. The uptake of NIC in fish is hypothesized to take place through the branchial epithelium, while the deposition of NIC has been observed in various tissues, such as the plasma, brain, liver, heart, muscle, and bile of teleost fishes [4]. It is hypothesized that NIC has the ability to disrupt the process of mitochondrial oxidative phosphorylation via uncoupling. In the case of tapeworms (*Cotugnia digonopora*), exposure to NIC led to the accumulation of lactate, decreased production of CO_2_, and reduced glycogen levels, indicating a potential impairment in aerobic ATP synthesis [13]. Multiple studies have demonstrated the evident toxicity of NIC exposure to aquatic organisms. For instance, the exposure of zebrafish embryos to NIC at environmentally relevant concentrations for a duration of 5 days yielded noteworthy impacts on both lipid metabolism and the endocrine system [2]. Furthermore, there have been reports indicating that NIC has the potential to induce disturbances in the metabolic processes and acid–base equilibrium of juvenile lake sturgeon [14]. In addition, due to the heightened rate of uncoupling brought about by NIC, it is anticipated that there will be an escalation in the presence of reactive oxygen species (ROS) within cellular environments. Our previous studies showed that NIC exposure was found to stimulate the formation of MDA content and impair antioxidant defense mechanisms in the liver and gut tissues of *Pelodiscus sinensis* [12]. We speculate that the damage of NIC to nontarget organisms is closely related to energy metabolism and oxidative damage.

As we all know, schistosomiasis has been demonstrated to have a notable prevalence and severity in the region of the Dongting Lakes, and the application of NIC as a primary agent in water serves to eradicate intermediate host snails, thereby effectively achieving the objective of disease eradication [12]. The black carp (*M. piceus*) is a notable indigenous fish species that inhabits a wide geographic area—specifically, region of the Dongting Lakes in Hunan Province, China [15]. Due to its distribution and characteristics, it serves as a valuable comparative model for simulating scenarios of exposure to NIC in the tributaries of Dongting Lakes. This allows for a deeper comprehension of the effects of this toxic substance on nontarget fishes. To date, there is a dearth of information pertaining to the impacts of waterborne NIC exposure on black carp. In this study, we conducted a chronic toxicology assessment to elucidate the effects of environmentally relevant concentrations of NIC on the enzyme activity and gene expression related to antioxidant and energy metabolism in black carp for the first time. Additionally, the concentrations of malonaldehyde, triphosphate, glucose, glycogen, lactic acid, cholesterol, and triglyceride, as well as the content of ATP, were also measured. Our objective was to establish a basis for future research on the effects of NIC on aquatic organisms and to contribute to the advancement of aquatic toxicology.

## 2. Materials and Methods

### 2.1. Ethics Statement

All animal procedures were carried out in strict accordance with the guidelines (license no. HNFI20221222), and approval was obtained from the animal welfare and ethics committee of Hunan Fisheries Science Institute (procedure approval 22 December 2022).

### 2.2. Niclosamide Preparation

Niclosamide (NIC, Formula: C_13_H_8_C_l2_N_2_O_4_, Cas: 50–65–7, purity ≥ 99%) was obtained from Shanghai Aladdin Biochemical Technology Co., Ltd. (Shanghai, China), and the solvent ethanol used in the experiment was from Xi-Long Scientific Co., Ltd. (Guangzhou, China). The preparation of the NIC exposure solution involved dissolving an NIC ingredient powder in hot ethanol, followed by dilution to a concentration of 2.0 g/L. According to our previous study and the range of environmentally relevant concentrations of NIC detected worldwide [7,12], the exposure concentrations were 10 μg/L and 50 μg/L in this study. The actual NIC level was determined through the utilization of high-performance liquid chromatography (HPLC) and was measured by Lanboru Co., Ltd. (Chengdu, China). To ensure the stability of the NIC concentrations throughout the experiment, water samples in each group were collected at 0, 7, 14, and 28 days of exposure and subsequently measured.

### 2.3. Animal Holding and Experimental Design

The healthy black carp (*n* = 90; mass: 38.2 ± 2.5 g; total length: 13.5 ± 1.2 mm) were sourced from an aquaculture facility affiliated with the Hunan Fisheries Science Institute (Changsha, China) and transported to the laboratory. The experimental fish were from the same family. All fish were reared in a 500 L freshwater system with oxygen-saturated water at a temperature of 28 °C and with a 12 h light–12 h dark photoperiod, and they were fed ad libitum twice per day (8:00 and 17:00) with commercial fish feed to apparent satiation. After being acclimated to the laboratory conditions for two weeks, the fish were subjected to experimental trials in which they were immersed in aqueous solutions containing NIC at concentrations of 0 (control group), 10, and 50 μg/L for 28 days. The water was refilled daily (to half the tank’s total volume) during the chronic test, and consistent water parameters were kept for each group. There were three 80 L tanks in each group with a density of 10 fish per tank. During the exposure period, the fish were fed twice a day, as before. The uneaten food was weighed after removing the feces every day with suction, but none of the treatment groups showed a difference in uneaten food. After the exposure period, no deaths were observed in any of the groups. Prior to sampling, a 24 h period of starvation was imposed on all fish, and three fish from each replicate tank were randomly selected and weighed to ascertain their weight gain. Subsequently, five other fish from each tank were randomly selected, anesthetized using MS-222 (Sigma-Aldrich, St. Louis, MO, USA), and then sacrificed on ice. Subsequently, the second gill arch, liver, and midgut tissue of the fish were promptly excised, cut into small pieces, rapidly frozen in liquid nitrogen, and preserved at a temperature of −80 °C for later examination of the tissue energy reserves (including ATP, acetyl-CoA, glycogen (Gln), glucose (Glu), lactate (LA), cholesterol (CHO), and triglyceride (TG)) and antioxidant metabolism (including superoxide dismutase (SOD), catalase (CAT), glutathione peroxidase (GPx), glutathione S-transferase (GST), and malondialdehyde (MDA)), as well as the gene expression and NIC concentrations. Owing to the small size of the fish, the five samples for each replicate tank were combined into a single Eppendorf tube after dissection, resulting in three duplicate samples per treatment group.

### 2.4. Determination of NIC Concentrations in Tissue

A total of 100 mg of each tissue sample (gill, liver, and midgut) was weighed and subsequently combined with 1 mL of acetonitrile for ultrasonication. Following this, anhydrous magnesium sulfate (0.3 g) was introduced, and the sample was thoroughly mixed using vortexing. The resultant mixture underwent centrifugation at a speed of 5000 r/min for a duration of 5 min, followed by extraction of 500 μL of the supernatant. The collected supernatant was quantified to determine the concentrations of NIC in the tissue using a high-performance liquid chromatography (HPLC) system, as described in our previous study [12].

### 2.5. Antioxidant Capacity Assays

The activity levels of superoxide dismutase (SOD), catalase (CAT), glutathione peroxidase (GPx), glutathione S-transferase (GST), and malonaldehyde (MDA) were assessed in the gill, liver, and gut samples of each fish from the different groups. This was done using standard kits obtained from Shanghai ZCIBIO Technology Co. Ltd., Shanghai, China, and by following the instructions provided by the manufacturer. All analyses were performed in triplicate.

### 2.6. Metabolic Parameter Assays

The concentrations of adenosine triphosphate (ATP), glucose (Glu), glycogen (Gln), lactic acid (LA), cholesterol (CHO), triglyceride (TG), and acetyl-CoA were measured using the corresponding commercial kits (Kit No. ZC-S0474, ZC-S0418, ZC-S0420, ZC-S0488, ZC-S0412, ZC-S0411, and ZC-S0393, respectively). The activity of lactate dehydrogenase (LDH), Na^+^/K^+^-ATPase, and Ca^+^/Mg^+^-ATPase was determined with the corresponding assay kits (Kit No. ZC-S0309, ZC-S0368, and ZC-S0369, respectively). All analyses were performed in triplicate.

### 2.7. Gene Expression Analysis

Total RNA was obtained from the gill, liver, and gut samples using a commercial RNA extraction kit (Cat. No. RE-03014, FOREGENE, Chengdu, China) according to the manufacturer’s instructions. The quantity and quality of RNA were calculated with a NanoDrop 2000 spectrophotometer (NanoDrop Technologies, Wilmington, DE, USA). Then, we checked the samples’ integrity on 1.0% agarose gels. The purified RNA was diluted to 15 ng/μL from the initial concentration and had an A260:280 ratio of 1.90–2.0. Then, two micrograms of RNA that was used as a template was immediately used to synthesize cDNA using the PrimeScript™ RT Reagent with a gDNA Eraser Kit (Cat. No. RE-03014, Foregene, Chengdu, China) according to the manufacturer’s instructions. A reaction mixture (20 µL) was prepared with 2 μL of cDNA template, 0.8 μmol/L of each primer, 6.4 μL of water, and 10 μL 2 × SYBR Green Master Mix kit (Takara, Japan), and the reaction was performed using the LightCycler^®^ 480 system (Roche, Switzerland). The primers of the target genes were summarized from previous studies, and the sequences provided by Sangon Biotech Co., Ltd. (Shanghai, China) were documented and are shown in Table 1. Each primer set was analyzed for melting curves to guarantee that a single product was amplified. The PCR procedure was performed as previously described [12]. The *β-actin* gene was employed as a reference gene in this experiment to determine the relative mRNA expression of target genes using the 2^−ΔΔCt^ method.

### 2.8. Statistical Analysis

The statistical software SPSS 22.0 (IBM, Chicago, IL, USA) was utilized to conduct all analyses. The data are presented as the mean ± standard deviation (SD). The homogeneity of variance was assessed using Levene’s equal-variance test, while the normal distribution was examined using the Shapiro–Wilk test for all of the data. Group means were compared using a one-way analysis of variance (ANOVA) followed by Tukey’s post-hoc test. Asterisks represent significant differences (* *p*-value < 0.05; ** *p*-value < 0.01; *** *p*-value < 0.001).

## 3. Results

### 3.1. NIC Exposure Reduced Fish Weight and Accumulated in Black Carp

**Figure 1:**
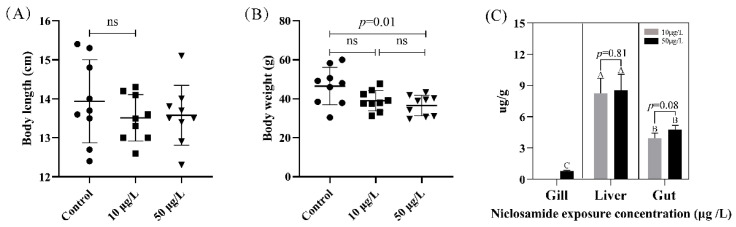
The growth performance and accumulation of NIC in black carp tissue. Note: Different capital letters indicate *p* < 0.05 in different tissues from the same group, and ns represents *p* > 0.05. (**A**) body length; (**B**) body weight; (**C**) NIC concentration in tissues.

As shown in Figure 1, no significant difference was observed in the body length (BL) of the black carp, although their body weight (BW) showed lower values with increasing NIC concentration compared to control group. At this time, the mean BW of the 50 μg/L NIC group exhibited a significant decrease in comparison to that of the control group (Figure 1B).

In addition, our chemical analysis demonstrated that there was a massive accumulation of NIC in the gill, liver, and midgut tissue of the black carp (Figure 1C). The liver exhibited the highest level of accumulation, followed by the gut, while the gill tissue displayed the least pronounced effects. Among these, the NIC concentrations from the 50 μg/L NIC groups were 0.78 ± 0.07 μg/L in the gill samples, and the NIC concentrations from the 10 and 50 μg/L NIC groups were 8.25 ± 1.19 and 8.56 ± 1.26 μg/L in the liver samples and 3.94 ± 0.39 and 4.78 ± 0.33 μg/L in the gut samples, respectively. The concentrations of NIC in the control group and in the gill tissues in the 10 μg/L NIC group were found to be below the detection limit according to the measurements that were taken. Interestingly, we found that the accumulation of NIC in all tested tissues except for the gills did not exhibit a significant increase with exposure to higher concentrations of NIC.

### 3.2. NIC Exposure Caused Damage to the Tight Junction in Black Carp

**Figure 2:**
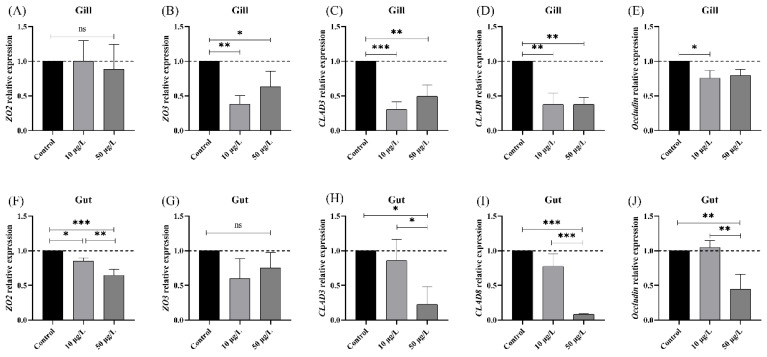
Expression of tight-junction genes in the gills and gut tissue after NIC exposure. Note: ns, *, **, and *** represent *p* > 0.05, *p* < 0.05, *p* < 0.01, and *p* < 0.001, respectively, between the different exposure groups. (**A**) *ZO2* in gill; (**B**) *ZO3* in gill; (**C**) *CLAD3* in gill, (**D**) *CLAD8* in gill; (**E**) *Occludin* in gill; (**F**) *ZO2* in gut; (**G**) *ZO3* in gut; (**H**) *CLAD3* in gut, (**I**) *CLAD8* in gut; (**J**) *Occludin* in gut.

As shown in Figure 2, the relative expression of *ZO2*, *ZO3*, *CLAD3*, *CLAD8,* and *Occludin* genes was detected in the gills and gut after NIC exposure. The results showed that the expression of the *CLAD3*, *CLAD8*, and *Occludin* genes was significantly downregulated in the gills and gut in the 10 and 50 μg/L NIC group. Regarding the ZO gene family, *ZO3* was significantly downregulated in the gills of all exposure groups (*p* < 0.05) but did not significantly change in the gut tissue (*p* > 0.05). In contrast, expression levels of *ZO2* significantly decreased in the gut of all exposure groups (*p* < 0.05) but did not significantly change in the gut tissue (*p* > 0.05).

### 3.3. NIC Exposure Decreased Antioxidant Ability in Black Carp

**Figure 3:**
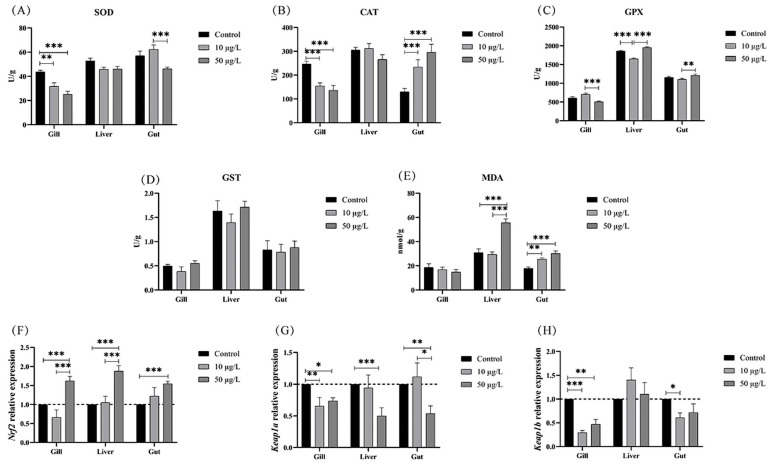
NIC exposure decreased the antioxidant ability in black carp. The activity of SOD (**A**), CAT (**B**), GPX (**C**), GST (**D**), and MDA (**E**) in each fish after NIC exposure. The mRNA expression of *Nrf2* (**F**), *Keap1a* (**G**), and *Keap1b* (**H**) in all tissues of the different groups. Note: *, **, and *** represent *p* < 0.05, *p* < 0.01, and *p* < 0.001, respectively, between the different exposure groups.

As shown in Figure 3, the results demonstrated that the SOD activity in the gill tissue was significantly lower in the 10 and 50 μg/L NIC groups than it was in the control group. The SOD activity in the gut tissue was significantly reduced in the 50 μg/L NIC group compared to that in the 10 μg/L NIC group (Figure 3A). In addition, NIC exposure also significantly decreased the CAT activity in the gills of the 10 and 50 μg/L NIC groups. Conversely, the activity of CAT in the gut tissue exhibited an opposite trend (Figure 3B). It was unexpected that no significant changes were shown in SOD and CAT activity in the liver tissue among the three groups. The levels of GPX activity in the gill tissue were found to be significantly reduced in the group that was exposed to 50 μg/L NIC compared to the group that was exposed to 10 μg/L NIC. Conversely, the trend observed in the gut tissue exhibited an opposite pattern. The GPX activity in the liver tissue was significantly lower in the 10 μg/L NIC group than it was in the control group and the 50 μg/L NIC group (Figure 3C). In addition, NIC exposure had no significant effect on the activity of GST in any of the examined tissues (Figure 3D). The findings of our study demonstrated that exposure to NIC significantly increased the concentration of MDA in the liver and gut tissues, particularly in the group that was exposed to 50 μg/L NIC (Figure 3E). These results provide evidence that environmentally relevant concentrations of NIC can induce oxidative damage.

In this study, it was observed that exposure to NIC resulted in an upregulation of the transcription level of *Nrf2* in comparison with that in the control group (Figure 3F). As expected, the gene expression of *keap1a* and *keap1b* exhibited a substantial decrease in nearly all groups that were exposed to NIC (Figure 3G,H). These findings indicated the activation of the cellular antioxidant response mechanism aimed at combatting oxidative damage. The aforementioned findings collectively indicated that exposure to NIC induced oxidative stress within the gill, liver, and gut tissues of black carp.

### 3.4. NIC Exposure Decreased the ATP Level and Inhibited ATPase Activity in Black Carp

**Figure 4:**
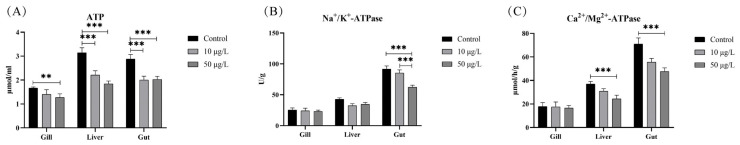
The ATP levels and ATPase activity in the gills, liver, and gut of black carp after NIC exposure at environmentally relevant concentrations. The data are expressed as means ± SD (n = 3). Note: (**A**) ATP level. (**B**) Na^+^/K^+^-ATPase activity. (**C**) Ca^+^/Mg^+^-ATPase activity. **, and *** represent *p* < 0.01 and *p* < 0.001, respectively, between the different exposure groups.

As shown in Figure 4, the levels of ATP exhibited a significant decrease in all of the examined tissues of the group that was exposed to a concentration of 50 μg/L NIC. Additionally, the liver and gut tissues of the group that was exposed to 10 μg/L NIC displayed a decreasing trend that was similar to that observed in the aforementioned group (Figure 4A). Furthermore, the activity of Na^+^/K^+^-ATPase was significantly inhibited in the gut of the groups exposed to 10 and 50 μg/L NIC, as depicted in Figure 4B. Conversely, the activity of Ca^2+^/Mg^2+^-ATPase was only notably inhibited in the liver and gut of the group exposed to 50 μg/L NIC (Figure 4C).

### 3.5. NIC Exposure Changed the Modes of Glucose and Lipid Metabolism in Black Carp

**Figure 5:**
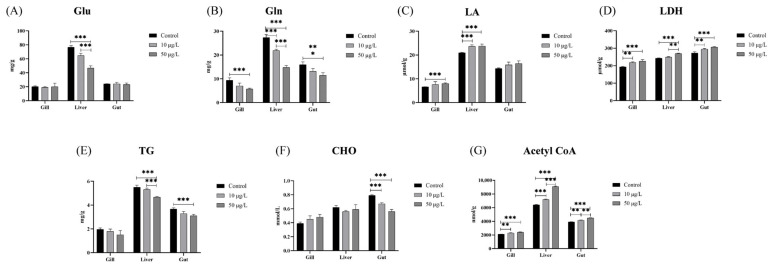
The levels of glucose (Glu), glycogen (Gln), lactic acid (LA), lactate dehydrogenase (LDH), triglyceride (TG), cholesterol (CHO), and acetyl-CoA in the gills, liver, and gut of black carp after NIC exposure. The data are expressed as means ± SD (n = 3). Note: *, **, and *** represent *p* < 0.05, *p* < 0.01, and *p* < 0.001, respectively, between the different exposure groups. (**A**) Glu; (**B**) Gln; (**C**) LA; (**D**) LDH; (**E**) TG; (**F**) CHO; (**G**) Acetyl CoA.

As shown in Figure 5, several metabolic parameters were measured in the gills, liver, and gut of black carp. The concentration of Glu in the liver tissue exhibited a significant decrease after exposure to NIC compared to the corresponding levels observed in the control group (Figure 5A). The levels of Gln in the liver tissue exhibited a dose-dependent decrease, with significant reductions being observed specifically in the 10 and 50 μg/L NIC groups. Meanwhile, there was a similar trend of change in the Gln levels in the gills and gut after NIC exposure (Figure 5B). In addition, the LA level in the gills and liver of the 50 μg/L NIC group was significantly decreased compared with that in the control group (Figure 5C). The activity of LDH exhibited a significant increase compared to that in the control group following exposure to a concentration of 50 μg/L NIC (Figure 5D). As shown in Figure 5E, the concentration of TG in the liver and gut of the NIC group at 50 μg/L exhibited a significant decrease compared to that in the control group, demonstrating a dose-dependent relationship. Similarly to the above results, the concentration of CHO in the gut tissue was significantly decreased in the 50 μg/L NIC group (Figure 5F). Meanwhile, the concentration of acetyl-CoA exhibited a dose-dependent increase in the presence of NIC, and statistically significant differences were observed (Figure 5G).

**Figure 6:**
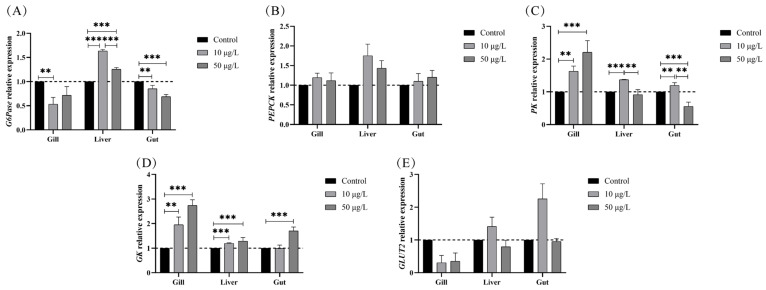
NIC exposure altered the gene expression of glucose metabolism enzymes in black carp. (**A**) *G6Pase*. (**B**) *PEPCK*. (**C**) *PK*. (**D**) *GK*. (**E**) *GLUT2*. The data are expressed as means ± SD (n = 3). Note: **, and *** represent *p* < 0.01 and *p* < 0.001, respectively, between the different exposure groups.

Notable changes in the gene expression of glucose metabolism enzymes in black carp compared to the control group are shown in Figure 6. According to our results, the expression of gluconeogenesis-related genes-specifically, *G6Pase*—exhibited a significant decrease in the gills and gut of fish following exposure to NIC. Conversely, the expression of *G6Pase* in the liver underwent a slight elevation in response to 10 μg/L NIC (Figure 6A). Moreover, the expression of glycolysis-related genes, including *PK* and *GK,* significantly increased after NIC exposure—except in the gut tissue—in the groups that were chronically exposed to 50 μg/L NIC (Figure 6C,D). However, there were no significant differences in the gene expression of *PEPCK* and *GLUT2* in any of the examined tissues from the NIC exposure groups (Figure 6B,D).

**Figure 7:**
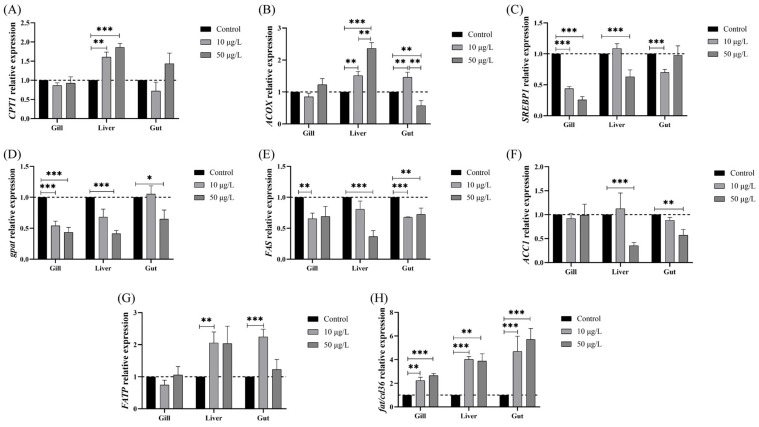
NIC exposure altered the gene expression of lipid metabolism enzymes in black carp. (**A**) *CPT1*. (**B**) *ACOX*. (**C**) *SREBP1*. (**D**) *GPAT*. (**E**) *FAS*. (**F**) *ACC1*. (**G**) *FATP*. (**H**) *Fat/cd36*. The data are expressed as means ± SD (n = 3). Note: *, **, and *** represent *p* < 0.05, *p* < 0.01, and *p* < 0.001, respectively, between the different exposure groups.

At the same time, we detected the mRNA levels of metabolism genes involved in β-oxidation (*CPT1* and *ACOX*), TG synthesis (*SREBP1*, *GPAT*, *FAS*, and *ACC1*), and fatty acid transportation (*FATP* and *Fat/cd36*) in order to investigate whether NIC exposure can affect lipid metabolism in carp. As shown in Figure 7, the relative expression of *CPT1* and *ACOX* in the gills was not drastically affected, but this expression was significantly increased in a dose-dependent manner after NIC exposure. In addition, the mRNA expression of *ACOX* in the gut was significantly increased in the 10 μg/L NIC group and decreased in the 50 μg/L NIC group (Figure 7A,B).

The expression level of *SREBP1* exhibited a noticeable reduction in the gills of the groups that were exposed to NIC, demonstrating a dose-dependent relationship. Additionally, a significant decrease in SREBP1 expression in comparison to that in the control group was observed in the gut in the 10 μg/L NIC group and in the liver in the 50 μg/L NIC group (Figure 7C). Moreover, the expression level of *GPAT* was noticeably reduced in a dose-dependent manner in the gills and liver of the NIC exposure groups, and its expression was also significantly decreased in the gut in the 50 μg/L NIC group when compared to that in the control group (Figure 7D). Compared to that in the control group, the mRNA expression of *FAS* was significantly downregulated in the gills in the 10 μg/L NIC group and in the liver in the 50 μg/L NIC group. The mRNA expression of *FAS* in the gut was lower in the NIC groups than in the control group (Figure 7E). Additionally, the gene expression of *ACC1* was markedly reduced in the liver and gut of the 50 μg/L NIC group (Figure 7F).

On the contrary, the mRNA expression of *FATP* exhibited a significant increase in both the liver and gut in the 10 μg/L NIC group. (Figure 7G). In addition, the expression level of *Fat/cd36* increased with a dose-dependent response in all tissues examined in the NIC groups, and it reached a maximum in the 50 μg/L NIC group (Figure 7H).

## 4. Discussion

Niclosamide (NIC) is a widely utilized molluscicide in China, and the extensive and prolonged application of NIC has given rise to pervasive concerns regarding environmental and biological safety, thereby garnering increasing human interest [12]. Meanwhile, NIC is often used as a veterinary anthelmintic in aquaculture, but its potential toxicity restricts its long-term usage to fish [5]. For instance, it was found that the exposure of zebrafish embryos to NIC at an environmentally realistic concentration (40 μg/L) could result in endocrine disruption [2]. NIC also caused toxic damage to the gill, brain, liver, and skeletal muscle tissues of *Lepomis macrochirus* [4]. However, previous studies pertaining to the toxicity of NIC have predominantly concentrated on its acute effects. The investigation of the chronic toxicity induced by NIC at environmentally relevant concentrations and its potential biological impacts on fish remain inadequately explored.

The first concern of our study was directed toward the epithelial tight junctions of the gills and gut tissue. These organs are in direct contact with water and play a crucial role in facilitating the absorption of NIC, making them the primary route for its uptake [4,21]. Typically, they serve as the foremost barrier against the entry of NIC and other xenobiotics [22,23,24]. In our results, it was observed that the expression of genes in the *ZO*, *CLAD*, and *Occludin* families, which are associated with epithelial tight junctions, were downregulated after NIC exposure. These transmembrane proteins (such as claudins and occludin) and cytoskeletal proteins (such as ZO) play an important role in the function of the mucosal barrier [25,26,27]. These findings imply that the integrity of the epithelial surface barrier was compromised in black carp. Interestingly, the degree of downregulation of tight-junction genes in the gut increased with the NIC exposure concentration, whereas the gills did not exhibit this trend. Moreover, the NIC concentration in black carp tissues significantly increased after chronic exposure, and the liver had the greatest accumulation. Recent studies showed that NIC accumulation and metabolism are primarily carried out in the liver after absorption, as this is the primary target organ [2,5]. This is similar to the results in our study. In addition, the levels of NIC accumulation in the gut were found to be higher than those in the gill tissue. This disparity could potentially be attributed to the more severe damage in the gut epithelial barrier, as further evidenced by the downregulation of tight-junction genes.

NIC has historically been regarded as an agent that disrupts the coupling of mitochondrial oxidative phosphorylation, which is primarily attributed to its possession of a phenol ring and its characteristics as a weak acid [21]. However, the increased rate of uncoupling may result in the generation of ROS [28]. Our prior research established that exposure to NIC elicits oxidative stress, which may subsequently result in the excessive production of reactive oxygen species (ROS), as indicated by the use of dihydroethidium (DHE) staining [12,29]. Oxidative stress represents a frequently encountered mechanism through which pollutants can elicit toxic effects [30]. In this study, the elevation of the MDA content meant that there was a significant level of lipid peroxidation, potentially resulting in substantial oxidative harm to the liver and gut or even disruption of its characteristic morphological integrity. In addition, SOD is postulated to serve as the primary mechanism for neutralizing superoxide radicals through its catalytic promotion of the disproportionation of H_2_O_2_ [31]. CAT has the ability to scavenge H_2_O_2_ in order to mitigate the peroxidation of unsaturated fatty acids within cell membranes [32]. In the present study, it was observed that the activity of SOD and CAT was suppressed, indicating that exposure to NIC hindered the antioxidant capacity. Interestingly, we found that the increase in CAT activity in the gut following NIC exposure may have been caused by an elevated production of reactive oxygen species, thereby potentially inducing the stimulation of CAT activity, which was similar to previous results in *Carassius auratus gibelio* [33]. Moreover, GPx exhibits a robust capacity to scavenge hydroxyl-peroxide-induced lipid peroxide and hydrogen peroxide, which are reactive oxygen species. This ability enables GPx to safeguard biomacromolecules and biofilm structures against peroxide-induced harm. Therefore, the observed inhibition of this activity provided evidence that NIC induces the impairment of antioxidant capacity through oxidative stress. Furthermore, Nrf2 is a crucial transcription factor that is sensitive to redox changes and regulates the transcription of genes implicated in antioxidant and detoxification processes via conjugation [34,35]. It assumes a central role in conferring cellular resilience against oxidative and electrophilic stress. Keap1 functions as a suppressor of Nrf2, whereby Nrf2 maintains its activity by associating with Keap1 under normal physiological conditions. Following the interaction with Keap1, Nrf2 translocates to the nucleus and forms a heterodimer with small Maf proteins within the nuclear environment [36]. In the presence of oxidative stress, Nrf2 exhibits a response by initiating and controlling a cascade of antioxidant proteins [34]. In this study, it is plausible to hypothesize that NIC may have induced the activation of Nrf2 signaling in a dose-dependent manner by inhibiting its ubiquitination and subsequent proteasomal degradation given that growth performance is often regarded as the most perceptible manifestation of organisms experiencing environmental stress [37].

A decrease in growth has been documented as a consequence of prolonged exposure to NIC, and a strong association exists between glucose and lipid metabolism [1,2,7]. Similarly, the findings of the current investigation demonstrated a noteworthy decline in BW among black carp in the 50 μg/L NIC group as the concentration of NIC exposure increased. This observation suggests that prolonged exposure to NIC adversely impacts the growth performance of fish. Moreover, our study also presented clear findings indicating evident disruptions in energy supply and glucose–lipid metabolism due to in vivo exposure to NIC, as demonstrated by the reduction in ATP production and perturbation of the mRNA expression of metabolic enzymes. A previous study indicated that NIC interferes with oxidative phosphorylation by the mitochondria, leading to decreased ATP production [38]. In our study, the observed phenomenon led to the activation of glycolysis as a means of fulfilling the energy demands of fish following exposure to NIC, which led to marked Glu and Gln depletion. During the process of glycolysis, Glu undergoes conversion into pyruvate (PA), resulting in the production of lactate as the final product. This metabolic pathway ultimately leads to the accumulation of LA, an increase in LDH activity, and a relatively low yield of ATP [39,40]. Glucose and lactate are integral components of the secondary stress response, and they exert a pivotal influence on energy metabolism and allocation [41]. In addition, Gln consumption, particularly in the liver, exhibited the most significant depletion in this study, suggesting that liver Gln is an important energy resource after exposure to NIC. Similar results have also been illustrated in juvenile lake sturgeon [14] and larval sea lamprey [8]. Moreover, the relative expression of several glucose metabolism genes was detected to reveal the overall impact of NIC on fish physiology. As is well known, Glu is rapidly metabolized into PA in the cytoplasm and is further metabolized into acetyl-CoA through key glycolytic genes, such as *GK* and *PK* [42]. After being exposed to NIC, it is theoretically anticipated that GK and PK, which serve as the initial and final stages in the glycolysis process, will exhibit heightened activity due to the exposure to NIC and the subsequent activation of the acute stress response. This can be attributed to the fact that glycolysis takes place instead, facilitating the mobilization of glucose to fulfill the energy requirements for detoxification. In addition, G6Pase, an enzyme that is crucial for liver gluconeogenesis, exhibits a rate-limiting role, while its mRNA expression level in the liver exhibits a strong correlation with gluconeogenic activity [43]. Therefore, our results in the present study indicated that the anaerobic glycolysis pathway was accelerated, and gluconeogenesis was inhibited in the response to NIC.

The main finding of our study was that coordination between glycose and lipid metabolism might be crucial for energy supply after NIC exposure in black carp. In this study, some of the genes that regulate *β*-oxidation (*CPT1* and *ACOX*), TG synthesis (*SREBP1*, *GPAT*, *FAS*, and *ACC1*), and fatty acid transportation (*FATP* and *Fat/cd36*) were noticeably disturbed by NIC exposure. In energy metabolism, the process of β-oxidation of mitochondrial fatty acids (FAs) plays a crucial role in the generation of ATP [44]. Importantly, *CPT1* and *ACOX* serve as the limiting factors in the β-oxidation of fatty acids [19]. *CPT1* is located within the outer mitochondrial membrane and plays a crucial role in governing the translocation of long-chain fatty acids into the mitochondria. On the other hand, some studies have indicated that *ACOX* specifically facilitates the β-oxidation of long-chain and very-long-chain fatty acids within peroxisomes [45]. This finding suggests that exposure to NIC may enhance the processes of lipolysis and fatty acid *β*-oxidation through the upregulation of the expression of genes associated with these metabolic pathways.

Furthermore, it is noteworthy that *SREBP1* serves as a pivotal transcription factor that governs the pathways involved in the synthesis of cholesterol, fatty acids, triacylglycerol, and phospholipids, as highlighted by a previous study [46]. Additionally, ACC1, an enzyme located in the cytosol, assumes a crucial function in the generation of malonyl-CoA and significantly contributes to the biosynthesis of long-chain fatty acids [45]. The process of fatty acid synthesis is initiated with the cleavage of citrate, which is derived from the tricarboxylic acid (TCA) cycle, into acetyl-CoA and oxaloacetate. This is followed by the carboxylation of acetyl-CoA, resulting in the production of malonyl-CoA. Subsequently, FAS assembles malonyl-CoA into long-chain fatty acids [47]. The downregulation of the mRNA expression levels of *SREBP1*, *GPAT*, *FAS*, and *ACC1* in the experimental group compared to the control group suggested that exposure to NIC significantly inhibited the synthesis of TG. A similar outcome was also documented in the embryonic stage of zebrafish that were acutely exposed to NIC at an environmentally realistic concentration [2]. Consistently, this result was also confirmed through histological analysis and the TUNEL assay in adult male zebrafish [7]. In addition, the cellular uptake of long-chain fatty acids is primarily facilitated by transporters, such as *FATP* and *FAT/CD36*, which play a significant role in the uptake and intracellular transport of fatty acids [16,48]. The findings of our study demonstrated a significant increase in the mRNA expression levels of *FATP* and *FAT/CD36* following exposure to NIC, thereby providing clear evidence of the enhanced uptake and transport of fatty acids, as well as the consequent expediting of their influx into tissue. Overall, it can be inferred that exposure to NIC may potentially enhance β-oxidation while inhibiting lipid synthesis in black carp based on the consistent findings of lower CHO and triglyceride (TG) levels in the NIC exposure groups.

## 5. Conclusions

In summary, the aforementioned findings suggest that exposure to NIC can result in compromised barrier function and oxidative stress, thereby facilitating the influx of harmful substances into the tissue. This exposure also hinders gluconeogenesis and TG synthesis while promoting glycolysis and lipolytic processes. Additionally, it significantly diminishes tissue energy reserves, including those of Gln, Glu, TG, and CHO, ultimately leading to substantial ATP consumption and a subsequent loss of body weight. As a result of the increasing demand for NIC and NIC-related pharmaceuticals, the dispersion of these compounds into the environment will expand, leading to a heightened severity of environmental contamination with NIC. Consequently, it is crucial to dedicate sustained attention to the potential ramifications of NIC exposure for wild aquatic organisms. Importantly, it is imperative to enhance our understanding of the biology and susceptibility of nontarget species to NIC. This knowledge is essential for equipping fishery managers and regulators with improved capabilities for effectively addressing this issue.

## Figures and Tables

**Table 1:** Sequence of primer pairs used in the real-time quantitative PCR reaction.

Target Gene	Abbreviation	Primer Sequences (5′-3′)	Accession Number	Annealing Temperature (°C)
Endogenous antioxidant defense				
NFE2-related nuclear factor 2	*NRF2*	Forward: TGTCCAAACACCAGCTCAA	cited by Ref. [16]	56
		Reverse: CGTACTCTAGGCCCACGAT		
Kelch-like-ECH-associated protein 1a	*Keap1a*	Forward: GGCTGCTCTGTGATCTGGTT	cited by Ref. [16]	56
		Reverse: TTCCTTGAAGTTGCTGGTGA		
Kelch-like-ECH-associated protein 1b	*Keap1b*	Forward: CCATCGGCATCGCCAACTT	cited by Ref. [16]	60
		Reverse: TGCGTAGCCACCTGACTGAA		
Physical barrier/Tight junction				
Zonula occludens-1	*ZO1*	Forward: CTCTTCACCACCACCATCAAC	cited by Ref. [16]	56
		Reverse: TCCGAGACCCAAACCAACT		
Zonula occludens-2	*ZO2*	Forward: GCCGTCTGGGTCTGTGAA	cited by Ref. [16]	58
		Reverse: CCGCCGTATCCTCGTAGTC		
Zonula occludens-3	*ZO3*	Forward: TGGCTCAGGAGAAGGGTG	cited by Ref. [16]	58
		Reverse: GCTGCTCGGACTGTTTGG		
Claudin3	*Claudin3*	Forward: GGAATGTGCGTGACCCTG	cited by Ref. [16]	56
		Reverse: TCCACAAGCCTTCATAGCG		
Claudin4	*Claudin4*	Forward: ACTGGGTGTTCTGGGAATCAAA	cited by Ref. [16]	58
		Reverse: GACGGGTACGAGGATGAGGA		
Claudin8	*Claudin8*	Forward: AGCGGTGCCCTGGAGATT	cited by Ref. [16]	60
		Reverse: CCACAAGCCTTCATAGCG		
Claudin15	*Claudin15*	Forward: GGTGTTCTGGGAATCAAATGC	cited by Ref. [16]	56
		Reverse: GCAGACGGGTACGAGGATG		
Occludin	*Occludin*	Forward: CCCGACGATGAGTTCCAG	cited by Ref. [16]	56
		Reverse: GAGGCCACGCATACGAAG		
Glucose metabolism				
Glucose-6-phosphatase	*G6Pase*	Forward: ATGCTTTCAGCTCACCGTCA	cited by Ref. [17]	58
		Reverse: TGTCGATGGGGACAGTTTGG		
Pyruvate kinase	*PK*	Forward: GCAGAGACGGAGCGTGATAA	cited by Ref. [18]	58
		Reverse: TTGCGACTTCCCAGAATCCC		
Phosphoenolpyruvate carboxykinase	*PEPCK*	Forward: CTGGCCTTGTAACCCAGAGA	cited by Ref. [17]	57
		Reverse: CACCATAACCACTGCCGAAG		
Glucokinase	*GK*	Forward: TGTGGTTGCCATGGTGAATG	cited by Ref. [17]	56
		Reverse: CTCTCCTTCAACCAGCTCCA		
Glucose transporter 2	*GLUT2*	Forward: AGATGGGCACACCTTACCT	cited by Ref. [17]	56
		Reverse: CAGACCACAGTACAGTCCCA		
Lipid metabolism				
Carnitine palmitoyltransferase 1	*CPT1*	Forward: TTTACGACGGACGGTTGC	MW048769	57
		Reverse: TGCTTGTTCTTCCCACGAC		
Acetyl-CoA carboxylase 1	*ACC1*	Forward: AGCCTCGGCACCACATAC	MW053372	56
		Reverse: GACCCTGAGAATCCAGAACC		
Acyl-coenzyme A oxidase	*ACOX*	Forward: AGGCTGGTCTGCTAAGTGTTC	MW053374	56
		Reverse: CATCTCATCGCGGTAGTCAA		
Sterol-regulatory-element-binding protein 1	*SREBP-1*	Forward: GAGAAACTGCCCATCAATC	MW048767	54
		Reverse: CCTCAACACTGCCGACTTA		
Fatty acid synthetase	*FAS*	Forward: AGAGCAACTACGGGTTCGC	MK986683	58
		Reverse: ACCTATCCAGCACCTCCAAAC		
Fatty acid transport protein	*FATP*	Forward: ATTCGGCGTATGTATCAGG	MK986680	60
		Reverse: CGAGCGTAAGAGGGAAGA		
Glycerol-3-phosphate acyltransferase	*Gpat*	Forward: ATCTGAGTACAACGCTCCC	cited by Ref. [19]	55
		Reverse: TCATCCAGTTTACGACGAAT		
Apolipoprotein A-1	*ApoA1*	Forward: TGAGAAGCTGACCAAGGACAT	cited by Ref. [20]	57
		Reverse: GTTCGGACTTCCTCCATGTG		
Fatty acid translocase	*FAT/CD36*	Forward: GGTCAACCCAGATAACCAGTG	cited by Ref. [19]	57
		Reverse: CTCATCAAAGTTCGGATTCAGT		
Housekeeping gene				
β-actin	*β-actin*	Forward: CCAGCAGATGTGGATTAGCA	KP185128	56
		Reverse: CAGTTTGAGTCGGCGTGA		

## Data Availability

This study are available on request from the corresponding author.
